# Determination and dietary intake risk assessment of 35 pesticide residues in cowpea (*Vigna unguiculata* [L.] Walp) from Hainan province, China

**DOI:** 10.1038/s41598-022-09461-w

**Published:** 2022-04-01

**Authors:** Qun Zhang, Chen Ma, Yun Duan, Xiaopeng Wu, Daizhu Lv, Jinhui Luo

**Affiliations:** 1 Analysis and Test Center, Chinese Academy of Tropical Agricultural Sciences, Haikou, 571101 Hainan China; 2 Hainan Provincial Key Laboratory of Quality and Safety for Tropical Fruit and Vegetable Products, Haikou, 571101 Hainan China; 3 Laboratory of Quality and Safety Risk Assessment for Tropical Products of Ministry of Agriculture and Rural Affairs, Haikou, 571101 Hainan China

**Keywords:** Plant sciences, Environmental sciences, Risk factors, Chemistry

## Abstract

The presence of pesticide residues in cowpea raises serious health concerns. In this study, a novel, sensitive, high-performance method was developed to simultaneously analyze the residues of 35 pesticides in cowpea samples from growing areas in the Hainan province of China, from November 2018 to June 2021. The method employs modified QuEChERS sample pretreatment coupled with gas chromatography-tandem mass spectrometry. The limits of quantification of the 35 pesticides in the cowpea matrix ranged from 1.0 to 8.0 μg/kg. Twenty-seven of the 35 pesticides were detected, twelve of which are banned for use on legumes in China. Residues for ten pesticides in 17.1% of the samples exceeded their MRLs, with the highest exceedance of 380% observed in difenoconazole. Moreover, 80.8% of the samples contained one or more pesticide residues, with the most frequently detected pesticide being chlorfenapyr with a detection rate of 46.3%. In addition, the pesticide triazophos was detected through different years and regions. Notably, the chronic dietary exposure risk (%ADI) of the detected pesticides, evaluated from the national estimated acceptable daily intake, was lower than 100% in Chinese people of different age groups.

## Introduction

Cowpea, *Vigna unguiculata* [L.] Walp, also known as bean, long bean, etc., is a vegetable of high nutritional value that has an important position and role in China's northern vegetable industry^1^. Hainan province is the main cowpea growing area of China's northern vegetable industry, with a yearly cowpea cultivation area of approximately 2.0 × 10^4^ hectares and an annual cowpea output exceeding 5.0 × 10^6^ tons (Statistical Bureau of Hainan Province, 2020)^2^. The high temperature and humidity in Hainan province are conducive to the occurrence of diseases and insect pests^3^. In addition, it is well known that cowpea is both a flower and fruit crop, which means its flowering period is also the harvest period^4^. Thus, there is a higher incidence of pest and disease outbreaks on cowpeas. As a result, farmers often spray various pesticides to improve the cowpea yield, the most common of which include fipronil, cyfluthrin, cyhalothrin, cypermethrin, pyridaben, and pyrimethanil. In this study, 35 pesticides, including prohibited pesticides, regulated pesticides that easily exceed the maximum residue limits (MRLs), and pesticides commonly applied to vegetable crops in China, were considered. Some of these pesticides are present in the samples through legal application to the crops while others are a result of illegal practices.

The widespread use of pesticides increases the chances for residues from the environment to enter the human body through food consumption. In recent years, multi-residue methods, including gas chromatography (GC)^5–9^, liquid chromatography (LC)^10–12^, spectral analysis^13^, immunoanalysis^14^ and electrochemical sensor technology^15,16^, have been used for detecting pesticide residues at trace concentrations in vegetables, fruits, and other food products. Moreover, GC or LC coupled to tandem mass spectrometry (GC–MS/MS or LC–MS/MS, respectively) has been used for the accurate, simultaneous determination of pesticide residues in agricultural and animal products^17–20^. Pesticide extraction in agricultural and animal products has been carried out using many different extraction techniques, namely solid-phase extraction (SPE)^8,21,22^, magnetic solid-phase extraction (MSPE)^23,24^; dispersive solid-phase extraction (DSPE)^17,25,26^, solid-phase micro-extraction (SPME)^27–29^, liquid–solid extraction (LSE)^22^, accelerated solvent extraction (ASE)^30^, ultrasonic assisted extraction (UAE)^31^, and the quick easy cheap effective rugged, and safe (QuEChERS) method^26,32,33^. Of these, the QuEChERS method has become the most common sample preparation method for the analysis of pesticide residues in fruits and vegetables, as is^26,32,34^ or with modifications^33,35,36^.

With these facts in mind, the aims of this study were: (1) to establish a rapid analysis method for the determination of 35 pesticides in cowpea by QuEChERS-gas chromatography-tandem mass spectrometry (QuEChERS-GC–MS/MS); (2) to analyze the residue levels of 35 pesticides in cowpea samples from the Hainan province in China; and (3) to preliminarily assess the chronic dietary intake risk of the pesticides detected in cowpea for different populations.

## Materials and methods

### Reagents and materials

Thirty-five pesticide residues were included in the analytical method, namely, acephate, azoxystrobin, chlordimeform, chlorfenapyr, chlorpyrifos-ethyl, coumaphos, cyfluthrin, cyhalothrin, cypermethrin, dicofol, difenoconazole, dimethoate, endosulfan, ethoprophos, fenitrothion, fenpropathrin, fenvalerate, fipronil, fluvalinate, isazophos, isocarbophos, isofenphos-methyl, malathion, methamidophos, omethoate, parathion-ethyl, parathion-methyl, pendimethalin, phorate, profenofos, pyridaben, pyrimethanil, sulfotep, terbufos, and triazophos.

Individual pesticide analytical standards were purchased from Dr. Ehrenstorfer GmbH (Germany), and stored in a freezer at − 20 °C. Acetonitrile and *n*-hexanes (HPLC grade) were purchased from Fisher Scientific (USA). Individual stock standard solutions were prepared at a concentration of 1000 mg/L with *n*-hexanes, while the working mixes were prepared from the original stock solutions. Finally, the working mixes were used to prepare the calibration curves and spiking tests.

The QuEChERS extraction kits, which include filter materials (4 g MgSO_4_, 1 g NaCl, 1 g Na_3_Citrate, and 0.5 g Na_2_HCitrate), a 50 mL tube, and ceramic homogenizers, were purchased from Agilent Technologies (Part Number: 5982-5650CH). The QuEChERS dispersive kits, which contain a 2 mL tube with 25 mg PSA, 2.5 mg GCB, and 150 mg MgSO_4_, were also purchased from Agilent Technologies (Part Number: 5982-5221).

A total of 574 samples of cowpea (*Vigna unguiculata* [L.] Walp) were purchased from different crop areas (including Sanya, Ledong, Lingshui, Wanning, Chengmai, and Haikou) in the Hainan province of China, from November 2018 to June 2021. At least 3 kg of cowpea pods per sample were bought, and subsequently sealed in a sterile polyethylene bag with a unique identification mark by NY/T 762-2004^37^. The blank samples were sealed in the same batch of sterile polyethylene bags. After collection, each sample was homogenized within 8 h and stored at − 20 °C until further analysis.

### Instruments and analytical conditions

The pesticides were analyzed using a Thermo Scientific™ Trace 1310-TSQ 9000 GC–MS/MS instrument. A TG-5SILMS glass capillary column (length 30 m, internal diameter 0.25 mm, and film thickness 0.25 μm) was used for the separation. The GC program was as follows: A total run time of 23.5 min; an initial column temperature of 70 °C held for 1 min, increased at 25 °C/min to 150 °C and held for 3 min, increased at 15 °C/min to 200 °C and held for 3 min, and finally increased at 20 °C/min to 300 °C and held for 5 min. The temperature of both the transfer line and ion trap was 300 °C, while the ionization energy was 70 eV. The injection port temperature was 260 °C, and 1 μL samples were injected in the splitless mode. Helium was used as a carrier at a flow rate of 1.2 mL/min. The mass spectrometer was operated in selected reaction monitoring (SRM) mode, as listed in Table 1, with the solvent delay set at 4 min.

**Table 1:** Retention times, quantitative and qualitative ions pair, collision energies for the tested pesticides in SRM mode.

Pesticide	T_r_ (min)	Quantitative ion pair, *m/z* (CE^a^, eV)	Qualitative ion pair, *m/z* (CE^a^, eV)
Acephate	7.41	94 > 64(8)	136 > 42.1(8), 136 > 94(12)
Azoxystrobin	21.49	344.1 > 156(34)	344.1 > 171.9(36), 344.1 > 329(14)
Chlordimeform	10.26	117.1 > 89.8(18)	181.1 > 140(16), 196 > 181.1(8)
Chlorfenapyr	16.57	136.9 > 102(12)	248.9 > 112(24), 248.9 > 137.1(18)
Chlorpyrifos-ethyl	14.30	196.7 > 107(36)	196.7 > 168.9(12), 313.9 > 257.9(12)
Coumaphos	19.27	209.9 > 119(22)	209.9 > 182(10), 226 > 163(18)
Cyfluthrin I–IV^a^	19.51 (peak1)	163 > 91.1(12)	163 > 127(6), 226 > 206.1(12)
	19.58 (peak2)	163 > 91.1(12)	163 > 127(6), 226 > 206.1(12)
	19.63 (peak3)	163 > 91.1(12)	163 > 127(6), 226 > 206.1(12)
	19.65 (peak4)	163 > 91.1(12)	163 > 127(6), 226 > 206.1(12)
Cyhalothrin I–II^a^	18.52 (peak1)	180.9 > 152(22)	197.1 > 141.1(10), 207.9 > 180.9(8)
	18.6 (peak2)	180.9 > 152(22)	197.1 > 141.1(10), 207.9 > 180.9(8)
Cypermethrin I–IV^a^	19.77 (peak1)	163 > 91.1(12)	163 > 127.1(6)
	19.84 (peak2)	163 > 91.1(12)	163 > 127.1(6)
	19.89 (peak3)	163 > 91.1(12)	163 > 127.1(6)
	19.92 (peak4)	163 > 91.1(12)	163 > 127.1(6)
Dicofol	14.70	111 > 74.9(12)	139 > 111(12), 250.9 > 139(12)
Difenoconazole I–II^a^	20.02 (peak1)	265 > 139(36)	265 > 202.1(16), 323 > 265(14)
	20.09 (peak2)	265 > 139(36)	265 > 202.1(16), 323 > 265(14)
Dimethoate	11.00	87 > 42.1(10)	93 > 63(8), 125 > 79(8)
Endosulfan I–II^a^	15.96 (peak1)	194.7 > 125(22)	194.7 > 159.4(8), 240.6 > 205.9(14)
	16.92 (peak2)	158.9 > 123(12)	194.7 > 159(8), 240.6 > 205.8(12)
Ethoprophos	10.02	157.9 > 96.9(16)	157.9 > 113.9(6), 200 > 158(6)
Fenitrothion	13.88	125 > 79(8)	277 > 109(16), 277 > 260(6)
Fenpropathrin	18.10	97.1 > 55.1(6)	181 > 151.9(22)
Fenvalerate	20.65	125 > 89(18)	167 > 89(32), 167 > 125(10)
Fipronil	15.20	366.9 > 212.9(28)	366.9 > 244.9(20), 368.8 > 214.9(30)
Fluvalinate I–II^a^	20.62 (peak1)	180.8 > 152.1(22)	250 > 55.1(16), 250 > 199.9(18)
	20.68 (peak2)	180.8 > 152.1(22)	250 > 55.1(16), 250 > 199.9(18)
Isazophos	11.96	118.9 > 76(18)	161 > 119(8), 161 > 146(6)
Isocarbophos	14.62	121.1 > 65(14)	136 > 69(30), 136 > 108(12)
Isofenphos-methyl	14.96	199 > 65(34)	199 > 121(10), 241.1 > 121.1(20)
malathion	14.13	92.8 > 63(8)	125 > 79(8),173.1 > 99(12)
Methamidophos	5.12	141 > 64(18)	141 > 79(20), 141 > 94.8(8)
Omethoate	9.55	110 > 79(10)	110 > 80(8), 156 > 110(8)
Parathion (ethyl)	14.51	109 > 81(10)	124.9 > 97(6), 291 > 109(12)
Parathion-methyl	13.11	124.9 > 47(12)	124.9 > 79(6), 263 > 109(12)
Pendimethalin	15.07	252.1 > 161(14)	252.1 > 162(8), 252.1 > 191.3(8)
Phorate	10.63	75 > 47(8)	121 > 65(8), 262 > 75(8)
Profenofos	16.26	296.7 > 268.9(10)	336.9 > 266.9(12), 336.9 > 308.9(8)
Pyridaben	19.29	147.1 > 117.1(20)	147.1 > 119.1(8), 147.1 > 132.1(12)
Pyrimethanil	11.80	198.1 > 117.9(30)	198.1 > 157.6(18), 198.1 > 182.9(14)
Sulfotep	10.41	202 > 145.9(10)	265.9 > 145.9(15), 322 > 202(10)
Terbufos	11.52	230.9 > 128.9(22)	230.9 > 174.9(12), 230.9 > 203(8)
Triazophos	17.17	161 > 105.7(12)	161 > 134.1(8), 172.1 > 77.1(25)

^a^Collision energy.

### Sample preparation and extraction

The QuEChERS method was chosen for the sample preparation in the initial experiment^35^. Briefly, a 10 g (accurate to 0.01 g) portion of milled sample was added into a 50 mL polytetrafluoroethylene (PTFE) centrifuge tube. Then, 20 mL of acetonitrile was added, and the samples were homogenized for 2 min. Subsequently, filter materials were added, and the samples were vigorously shaken for 1 min. The extract was then centrifuged (10,000 rpm) for 5 min. Next, 1.5 mL of the supernatant (acetonitrile phase) was transferred to a 2 mL centrifuge tube containing 25 mg PSA, 2.5 mg GCB, and 150 mg MgSO_4_ and vigorously shaken for 1 min. The tube was then centrifuged (12,000 rpm) for a further 5 min. Finally, the acetonitrile extracts were filtered through a 0.22 µm PTFE filter and analyzed by GC–MS/MS.

### Method accuracy

Method accuracy was performed on five replicates of cowpea extracts at each of the three spiking levels 50, 100, and 250 μg/kg. The reproducibility was evaluated by performing another set of recovery tests, under the same conditions, after the cowpea samples were analyzed. For these tests, a blank sample was used and spiked with the same standard levels as those used in the previous recovery studies. The limits of detection quantification (LOQ) were set at the minimum concentration that could be quantified with acceptable values of recovery (70–120%) and relative standard deviation (RSD ≤ 20%), as advised by the European Union SANTE/12682/2019 regulatory guidelines^38^. The linearity of both solvent and matrix-matched calibration curves was assessed by the injection of 2.5, 5, 10, 50, 100, 250, and 500 μg/L calibration points (seven points). The matrix effect (ME) was assessed by comparing the corresponding matrix-matched slopes with the solvent calibration slopes as follows:

1 $$ME = \frac{A - B}{B} \times 100\%$$

where A is the slope in the matrix, and B is the slope in the solvent. At values > 0, the ME represents the enhancement of the matrix response to analysis; at values < 0, the matrix has an inhibitory effect on the response of the analyte; and at ME = 0, there is no matrix effect. At ME < − 50% or ME > 50%, the matrix interference degree is strong; at − 50% ≤ ME < − 20% or 20% < ME ≤ 50%, the interference degree of the matrix is moderate; and at − 20% ≤ ME ≤ 20%, the matrix interference is low.

### Dietary intake risk assessment

The chronic dietary exposure risk (%ADI) of the pesticide residues in people of different age groups was calculated as follows:

2 $$\% ADI = \frac{{C_{i} \times F}}{bw \times ADI} \times 100$$

where the %ADI is the chronic exposure risk^39^, F (kg) is the average daily intake of a certain food in China (Table 2), bw (kg) is the average body weight of the Chinese of different age groups (Table 2), C_i_ (mg/kg) is the average concentration of pesticide residues in cowpea from the Hainan province, China (Table 3), and ADI (mg/kg·bw) is the acceptable daily intake of detectable pesticide residues (Table 3). At %ADI < 100, the risk is acceptable and does not constitute a health threat in the long term, while %ADI values > 100 pose an unacceptable risk^39^.

**Table 2:** Average cowpea intake and body weights of the 10 age/sex groups in China.

Age	Sex	Average body weight (kg)	Average cowpea intake (g)
2–7	–	17.9	10.53
8–12	–	33.1	15.5
13–19	M^a^	56.4	18.6
	F^b^	50.0	19.1
20–50	M^a^	63.0	21.6
	F^b^	56.0	20.2
51–65	M^a^	65.0	22.2
	F^b^	58.0	20.0
> 65	M^a^	59.5	19.4
	F^b^	52.0	18.0

^a^Male; ^b^female.

**Table 3:** Chronic dietary exposure risk (%ADI) of pesticide residue in Hainan cowpea samples among different subgroups based on average concentration.

Pesticide	Min–Max (mg/kg)	MRLs^a^ (mg/kg)	ADI^b^ (mg/kg bw)	Average (mg/kg)	Chronic dietary exposure risk (%ADI) of different subgroups (age)									
					2–7	8–12	13–19		20–50		51–65		> 65	
							M	F	M	F	M	F	M	F
Acephate	0.004–0.41	Banned^a1^	0.03	0.0119	0.0233	0.0185	0.0131	0.0151	0.0136	0.0142	0.0135	0.0137	0.0129	0.0137
azoxystrobin	0.004–0.36	3^a3^	0.2	0.0124	0.0036	0.0029	0.0020	0.0024	0.0021	0.0022	0.0021	0.0021	0.0020	0.0021
Chlordimeform	0.012–1.3	Banned^a1^	0.001	0.0398	2.3409	1.8634	1.3123	1.5201	1.3644	1.4283	1.3591	1.3722	1.2975	1.3775
Chlorfenapyr	0.004–1.6	2^a3^	0.03	0.2069	0.4056	0.3229	0.2274	0.2634	0.2364	0.2475	0.2355	0.2378	0.2248	0.2387
Chlorpyrifos-ethyl	0.004–2.7	Banned^a1^	0.01	0.0276	0.1626	0.1294	0.0912	0.1056	0.0948	0.0992	0.0944	0.0953	0.0901	0.0957
Coumaphos	0.006–0.018	Banned^a1^	0.003	0.0001	0.0011	0.0009	0.0006	0.0007	0.0007	0.0007	0.0007	0.0007	0.0006	0.0007
Cyfluthrin	0.004–1.9	0.5^a3^	0.04	0.0181	0.0266	0.0212	0.0149	0.0173	0.0155	0.0162	0.0155	0.0156	0.0148	0.0157
Cyhalothrin	0.004–3.5	0.2^a2^	0.02	0.0174	0.0512	0.0408	0.0287	0.0332	0.0298	0.0312	0.0297	0.0300	0.0284	0.0301
Cypermethrin	0.004–1.3	0.5^a2^	0.02	0.1392	0.4094	0.3259	0.2295	0.2658	0.2386	0.2498	0.2377	0.2400	0.2269	0.2409
Dicofol	0.005–0.21	Banned^a1^	0.002	0.0007	0.0206	0.0164	0.0115	0.0133	0.0120	0.0125	0.0119	0.0120	0.0114	0.0121
Difenoconazole	0.004–1.9	0.5^a3^	0.01	0.0959	0.5639	0.4489	0.3161	0.3662	0.3286	0.3441	0.3274	0.3305	0.3125	0.3318
Fenitrothion	0.059–0.96	0.5^a2^	0.006	0.0018	0.0174	0.0139	0.0098	0.0113	0.0101	0.0106	0.0101	0.0102	0.0096	0.0102
Fenpropathrin	0.002–1.3	1^a3^	0.03	0.0163	0.0320	0.0254	0.0179	0.0207	0.0186	0.0195	0.0185	0.0187	0.0177	0.0188
Fenvalerate	0.010–1.1	3^a3^	0.02	0.0241	0.0709	0.0565	0.0398	0.0461	0.0413	0.0433	0.0412	0.0416	0.0393	0.0417
Fipronil	0.006–0.32	Banned^a1^	0.0002	0.0017	0.4941	0.3933	0.2770	0.3209	0.2880	0.3015	0.2869	0.2896	0.2739	0.2908
Fluvalinate	0.011–0.032	0.5^a3^	0.005	0.0002	0.0027	0.0021	0.0015	0.0017	0.0016	0.0016	0.0016	0.0016	0.0015	0.0016
Isazophos	0.004–0.25	Banned^a1^	0.00005	0.0022	2.5617	2.0392	1.4361	1.6635	1.4930	1.5630	1.4873	1.5016	1.4199	1.5074
Isocarbophos	0.004–0.008	Banned^a1^	0.003	0.00004	0.0008	0.0007	0.0005	0.0005	0.0005	0.0005	0.0005	0.0005	0.0005	0.0005
Malathion	0.010–0.048	2^a3^	0.3	0.0001	0.0000	0.0000	0.0000	0.0000	0.0000	0.0000	0.0000	0.0000	0.0000	0.0000
Methamidophos	0.008–0.48	Banned^a1^	0.004	0.0017	0.0246	0.0196	0.0138	0.0159	0.0143	0.0150	0.0143	0.0144	0.0136	0.0145
Parathion-methyl	0.004–0.018	Banned^a1^	0.003	0.0003	0.0065	0.0052	0.0036	0.0042	0.0038	0.0040	0.0038	0.0038	0.0036	0.0038
Pendimethalin	0.010–0.016	0.05^a4^	0.1	0.0001	0.0000	0.0000	0.0000	0.0000	0.0000	0.0000	0.0000	0.0000	0.0000	0.0000
Profenofos	0.004–1.4	10^a3^	0.03	0.0687	0.1348	0.1073	0.0756	0.0875	0.0786	0.0822	0.0783	0.0790	0.0747	0.0793
Pyridaben	0.004–2.2	2^a3^	0.01	0.0453	0.2666	0.2122	0.1495	0.1731	0.1554	0.1627	0.1548	0.1563	0.1478	0.1569
Pyrimethanil	0.004–0.33	2^a3^	0.2	0.0013	0.0004	0.0003	0.0002	0.0002	0.0002	0.0002	0.0002	0.0002	0.0002	0.0002
Sulfotep	0.008–0.008	Banned^a1^	0.001	0.00001	0.0008	0.0007	0.0005	0.0005	0.0005	0.0005	0.0005	0.0005	0.0005	0.0005
Triazophos	0.006–0.79	Banned^a1^	0.001	0.0072	0.4214	0.3354	0.2362	0.2736	0.2456	0.2571	0.2446	0.2470	0.2335	0.2479

^a^Maximum residue limits: ^a1^The pesticide is banned on legumes in China; ^a2^The Chinese national standard GB/T 2763–2021; ^a3^The maximum residue limits of pesticide in vegetable routine monitoring in 2015; ^a4^The maximum residue limits of pesticide in European Commission (https://ec.europa.eu/food/plant/pesticides/eu-pesticides-database/mrls/?event=search.pr).

^b^Acceptable daily intakes (ADIs) was referred to the Chinese national standard GB/T 2763-2021.

## Results and discussion

### Matrix effects (ME) and method accuracy

The complexity of a vegetable matrix may affect the analysis by inhibiting or enhancing the response, thus affecting the accuracy, selectivity, and sensitivity of the method^35,36^. Thus, if the signal suppression or enhancement exceeds 20%, the ME should be addressed in the calibration^38^. In this study, 11.4% of the 35 pesticides showed negligible ME (< 20%), 48.6% of them showed medium ME (20% < ME < 50%), while 40.0% exhibited strong signal suppression (> 50%) (Table 4). It has been reported that 98% of the 218 compounds analyzed by GC–MS/MS presented significant enhancement caused by the co-extraction of the matrix components^36^. Conversely, only 7% of the pesticides showed signal suppression in complex herb matrices^40^. According to Krynitsky et al., even after comprehensive extensive sample extraction, there were still sufficient co-extraction compounds that could result in signal suppression or signal enhancement, adversely affecting the quantitative analysis^41^. Therefore, in this study, to avoid ME, the results were quantified by an external standard method using matrix-matched calibration curves.

**Table 4:** The results of method accuracy for this study.

Pesticide	Calibration curve equations	R^2a^	LOQ^b^ (μg/kg)	Average recoveries (%) ± RSD^c^ (%)							ME^d^ (%)
				LOQ	Treatment 1^e^			Treatment 2^f^			
					50 μg/kg	100 μg/kg	250 μg/kg	50 μg/kg	100 μg/kg	250 μg/kg	
Acephate	*Y* = *5.212e*^*4*^*X* − *1.546e*^*3*^	0.9999	8.0	96.8 ± 7.4	92.3 ± 6.7	86.1 ± 3.2	92.8 ± 3.3	95.0 ± 8.5	80.6 ± 9.5	82.2 ± 4.1	57.8
Azoxystrobin	*Y* = *1.705e*^*4*^*X* + *3.256e*^*4*^	0.9999	1.0	105.2 ± 2.1	102.6 ± 4.1	113.6 ± 3.7	113.8 ± 1.4	91.0 ± 9.8	93.1 ± 3.7	98.7 ± 1.6	60.4
Chlordimeform	*Y* = *3.537e*^*4*^*X* + *2.574e*^*5*^	0.9999	6.0	98.3 ± 1.6	96.2 ± 8.4	92.9 ± 9.1	110.5 ± 1.6	87.6 ± 7.2	99.9 ± 6.7	92.1 ± 3.5	23.03
Chlorfenapyr	*Y* = *7.664e*^*3*^*X* + *3.752e*^*4*^	0.9999	6.0	87.5 ± 6.5	88.0 ± 4.1	96.8 ± 4.6	102.3 ± 0.5	81.6 ± 4.9	86.9 ± 1.7	86.3 ± 8.6	35.5
Chlorpyrifos-ethyl	*Y* = *1.744e*^*5*^*X* + *1.16e*^*5*^	1	1.0	112.7 ± 6.1	114.0 ± 8.5	109.1 ± 2.4	103.7 ± 1.0	99.6 ± 5.2	95.9 ± 2.3	89.9 ± 2.2	38.2
Coumaphos	*Y* = *1.695e*^*4*^*X* + *2.614e*^*4*^	0.9998	5.0	101.4 ± 6.7	96.2 ± 4.1	99.3 ± 5.2	102.2 ± 2.4	87.9 ± 3.7	85.8 ± 5.2	90.3 ± 9.6	75.88
Cyfluthrin I–IVa	*Y* = *7.225e*^*4*^*X* − *4.985e*^*4*^	0.9999	5.0	82.0 ± 8.9	96.6 ± 5.1	96.1 ± 1.5	96.0 ± 1.4	100.8 ± 2.7	91.5 ± 1.7	86.1 ± 2.5	37.49
CyhalothrinI–II^a^	*Y* = *2.142e*^*5*^*X* + *4.511e*^*5*^	0.9998	5.0	70.6 ± 8.4	77.6 ± 2.4	90.8 ± 2.4	101.9 ± 1.2	82.6 ± 3.6	79.0 ± 2.9	88.8 ± 2.4	66.8
CypermethrinI–IV^a^	*Y* = *9.391e*^*4*^*X* + *6.153e*^*5*^	0.9999	2.0	86.0 ± 7.7	98.0 ± 10.0	99.9 ± 6.5	89.3 ± 3.0	114.0 ± 4.0	96.6 ± 3.2	91.1 ± 9.0	41.88
Dicofol	*Y* = *6.647e*^*3*^*X* + *1.245e*^*3*^	0.9999	5.0	118.1 ± 4.8	110.5 ± 4.2	107.9 ± 4.5	101.3 ± 3.0	100.6 ± 11.6	90.0 ± 9.3	95.6 ± 4.7	48.47
DifenoconazoleI–II^a^	*Y* = *1.755e*^*5*^*X* + *2.396e*^*5*^	0.9999	1.0	79.9 ± 1.0	90.8 ± 2.8	100.3 ± 3.2	112.6 ± 2.0	94.2 ± 4.7	113.5 ± 2.3	99.3 ± 3.2	65.57
Dimethoate	*Y* = *3.456e*^*4*^*X* − *8.066e*^*4*^	0.9999	5.0	110.1 ± 8.8	112.2 ± 5.2	114.4 ± 3.0	97.9 ± 1.5	100.0 ± 4.2	107.0 ± 7.2	85.9 ± 4.0	64.1
EndosulfanI–II^a^	*Y* = *2.1338e*^*4*^*X* − *2.136e*^*3*^	0.9999	5.0	108.9 ± 5.8	105.9 ± 1.2	97.0 ± 0.9	96.8 ± 0.4	88.4 ± 2.0	85.5 ± 1.3	84.1 ± 1.7	19.71
Ethoprophos	*Y* = *7.035e*^*4*^*X* − *6.293e*^*4*^	0.9999	1.0	113.4 ± 6.2	102.9 ± 0.9	106.5 ± 1.1	99.7 ± 1.6	95.4 ± 3.8	99.9 ± 2.1	89.8 ± 2.0	54.34
Fenitrothion	*Y* = *4.158e*^*4*^*X* + *5.831e*^*5*^	0.9999	6.0	80.2 ± 8.6	93.6 ± 7.4	104.0 ± 5.9	101.2 ± 1.7	97.7 ± 8.5	94.4 ± 3.7	100.4 ± 3.1	81.81
Fenpropathrin	*Y* = *1.811e*^*5*^*X* + *4.155e*^*5*^	0.9991	6.0	82.1 ± 7.6	94.5 ± 2.0	95.2 ± 1.6	111.3 ± 1.9	80.7 ± 3.6	87.2 ± 2.5	95.0 ± 1.2	28.83
Fenvalerate	*Y* = *1.156e*^*4*^*X* + *2.08e*^*4*^	1	3.0	117.1 ± 7.5	103.1 ± 6.9	108.5 ± 6.4	91.7 ± 2.6	115.1 ± 6.1	113.6 ± 5.9	105.9 ± 1.9	42.07
Fipronil	*Y* = *6.462e*^*4*^*X* − *2.177e*^*5*^	0.9995	3.0	111.6 ± 6.8	101.4 ± 1.0	94.2 ± 1.3	95.6 ± 0.7	105.6 ± 7.5	88.0 ± 1.3	85.1 ± 2.3	63.22
FluvalinateI–II^a^	*Y* = *5.759e*^*4*^*X* − *2.698e*^*5*^	0.999	5.0	80.2 ± 9.5	98.1 ± 9.7	84.5 ± 1.7	83.2 ± 5.7	90.5 ± 2.7	79.8 ± 2.8	78.0 ± 10.1	74.62
Isazophos	*Y* = *3.008e*^*5*^*X* − *2.941e*^*5*^	1	3.0	115.1 ± 8.7	107.2 ± 1.3	102.7 ± 2.0	108.5 ± 0.8	110.8 ± 8.4	94.9 ± 2.4	108.4 ± 1.4	11.08
Isocarbophos	*Y* = *6.758e*^*4*^*X* − *1.244e*^*5*^	0.9999	2.0	119.1 ± 8.4	108.5 ± 0.8	102.7 ± 4.7	99.3 ± 1.2	94.9 ± 4.6	97.8 ± 2.3	88.0 ± 1.6	49.25
Isofenphos-methyl	*Y* = *1.054e*^*5*^*X* − *1.818e*^*5*^	0.9999	2.0	114.3 ± 7.6	104.3 ± 3.7	98.3 ± 3.2	96.3 ± 0.9	93.0 ± 3.5	90.3 ± 3.1	85.0 ± 1.1	43.15
malathion	*Y* = *1.378e*^*5*^*X* + *5.087e*^*6*^	0.9997	2.0	80.2 ± 7.4	91.4 ± 8.1	91.0 ± 7.3	96.8 ± 4.4	95.8 ± 6.1	104.5 ± 7.7	106.3 ± 5.9	43.77
Methamidophos	*Y* = *1.278e*^*4*^*X* − *2.398e*^*4*^	0.9998	3.0	84.2 ± 9.3	94.6 ± 2.2	91.8 ± 1.8	86.3 ± 2.5	84.8 ± 4.3	92.2 ± 4.1	88.8 ± 3.4	35.38
Omethoate	*Y* = *2.033e*^*5*^*X* + *5.787e*^*5*^	0.9995	5.0	116.1 ± 9.6	110.1 ± 8.6	95.5 ± 3.1	93.2 ± 1.7	93.5 ± 4.2	84.8 ± 5.6	80.1 ± 3.9	− 68.57
Parathion (ethyl)	*Y* = *5.402e*^*4*^*X* − *1.72e*^*5*^	0.9997	3.0	118.1 ± 2.7	101.7 ± 3.8	97.1 ± 2.7	94.5 ± 1.2	92.5 ± 8.6	89.1 ± 2.6	88.3 ± 2.2	76.31
Parathion-methyl	*Y* = *8.471e*^*4*^*X* − *1.55e*^*5*^	0.9999	3.0	80.2 ± 8.9	99.8 ± 3.2	99.1 ± 1.6	98.2 ± 1.5	81.6 ± 4.6	88.9 ± 2.4	91.0 ± 2.0	75.82
Pendimethalin	*Y* = *3.94e*^*4*^*X* − *1.602e*^*5*^	0.9993	7.5	82.1 ± 4.9	97.9 ± 3.0	91.9 ± 2.0	87.5 ± 1.3	96.9 ± 9.2	86.6 ± 1.6	91.9 ± 2.8	45.01
Phorate	*Y* = *3.409e*^*4*^*X* − *1.537e*^*4*^	1	3.0	117.8 ± 7.0	116.5 ± 2.0	104.7 ± 0.7	100.8 ± 1.5	100.8 ± 1.6	97.5 ± 1.8	92.7 ± 0.8	36.36
Profenofos	*Y* = *9.018e*^*4*^*X* + *2.566e*^*4*^	1	3.0	115.1 ± 8.5	108.4 ± 1.8	103.3 ± 2.0	100.1 ± 1.2	90.9 ± 2.2	86.8 ± 2.2	86.8 ± 2.2	81.08
Pyridaben	*Y* = *2.989e*^*5*^*X* + *7.01e*^*5*^	0.9998	2.0	78.6 ± 9.1	85.5 ± 4.3	96.4 ± 2.2	103.8 ± 1.9	104.0 ± 4.4	100.2 ± 9.5	119.1 ± 4.2	55.19
Pyrimethanil	*Y* = *9.968e*^*4*^*X* − *9.818e*^*3*^	1	1.0	115.3 ± 7.2	100.7 ± 0.8	96.9 ± 1.4	94.9 ± 0.7	104.4 ± 2.0	87.4 ± 1.3	83.1 ± 2.3	35.95
Sulfotep	*Y* = *7.091e*^*4*^*X* − *4.793*^*3*^	1	6.0	116.8 ± 6.8	108.7 ± 2.0	101.4 ± 1.1	101.2 ± 1.7	89.2 ± 6.5	89.8 ± 2.5	88.0 ± 2.5	37.8
Terbufos	*Y* = *1.85e*^*5*^*X* + *7.66e*^*2*^	1	3.0	117.3 ± 4.7	114.7 ± 1.9	105.2 ± 1.7	101.9 ± 1.2	89.1 ± 3.7	94.8 ± 2.1	92.8 ± 1.8	34.25
Triazophos	*Y* = *2.091e*^*4*^*X* + *2.213e*^*4*^	0.9999	1.0	113.3 ± 8.4	103.6 ± 3.5	96.6 ± 3.0	96.2 ± 0.9	93.4 ± 1.3	90.6 ± 2.2	85.2 ± 1.2	60.72

^a^Determination coefficient, ^b^limit of quantification, ^c^relative standard deviations, ^d^Matrix effect, ^e^Tests were performed in three levels, five replicates each before the cowpea samples were analysed, ^f^Tests were performed in three levels, five replicates each after the cowpea samples were analysed.

Method validation results are shown on Table 4. The table shows that the average recoveries of the 35 pesticides ranged between 77.6 and 119.1% when the spiked levels were 50, 100, and 250 µg/kg, with relative standard deviations (RSDs) in the range of 0.4–11.6%. In addition, there was no significant difference between the cowpea samples before and after analysis. The calibration curves of the 35 pesticides ranged from 2.5 to 500 µg/L, and the correlation coefficients all exceeded 0.9990. In addition, the LOQs ranged from 1.0 to 8.0 μg/kg, which are lower than the Chinese MRLs (Table 3). According to the Guidance SANTE/12682/2019^38^, this method meets the requirements for the determination of the selected pesticides in the cowpea samples.

### Verification and analysis of cowpea samples

**Figure 1:**
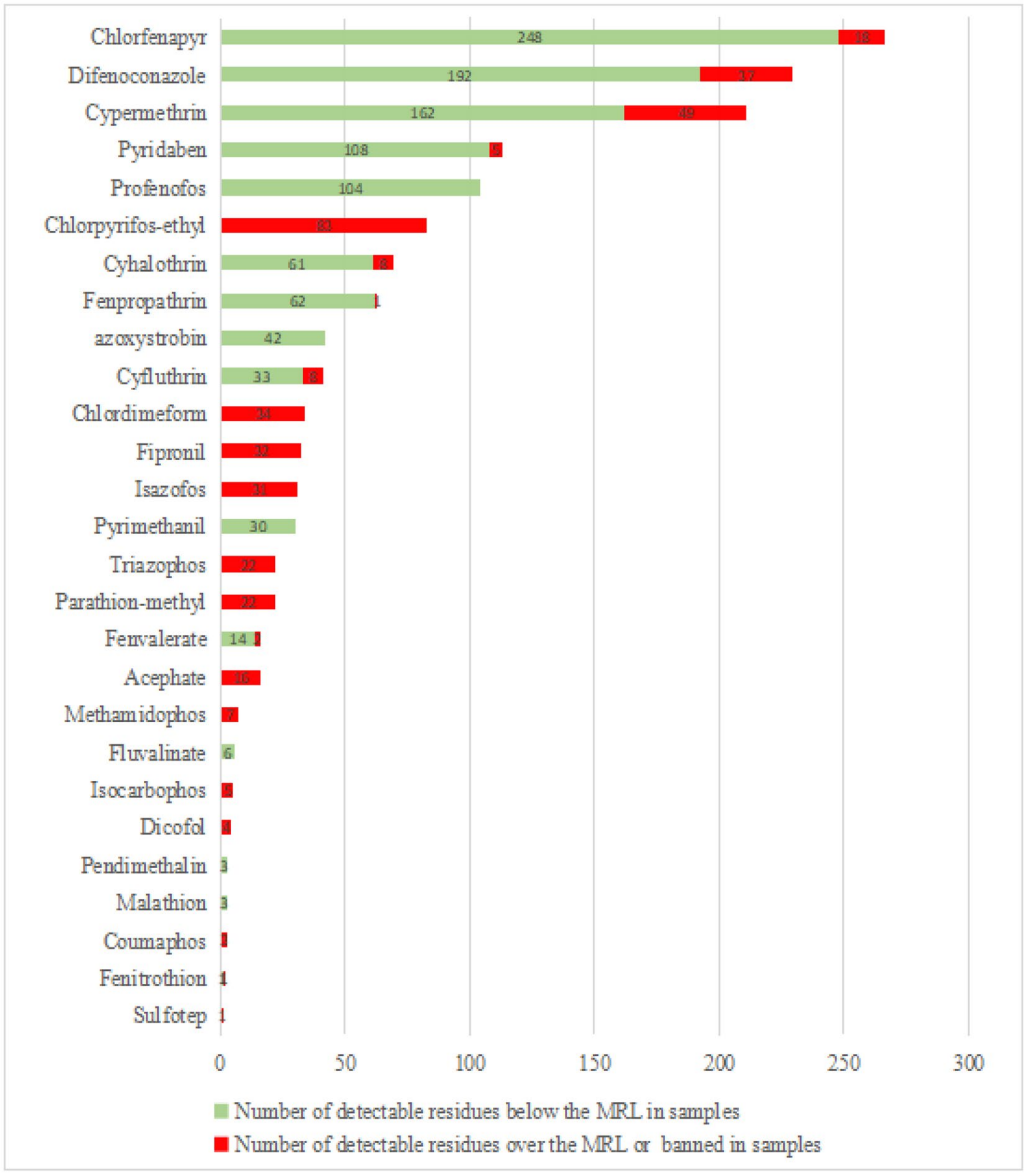
The pesticide in the detected samples.

The validated analytical method was used to analyse 35 pesticide residues in 574 cowpea samples collected from markets, supermarkets, and planting bases from Hainan province, China. As shown in Fig. 1, 27 of the 35 pesticides were detected at least once. There were eight pesticides with a detection rate > 10%: The most frequently detected pesticide was the insecticide chlorfenapyr (46.3%), followed by the fungicide difenoconazole (39.9%), the insecticide cypermethrin (36.8%), the acaricide pyridaben (19.7%), and subsequently the insecticides profenofos (18.1%), chlorpyrifos-ethyl (14.5%), cyhalothrin (12.0%), and fenpropathrin (11.0%). According to the list of prohibited pesticides on legumes in China, twelve banned pesticides were detected, namely, in decreasing order, chlorpyrifos-ethyl (14.5%), chlordimeform (5.9%), fipronil (5.6%), isazophos (5.4%), parathion-methyl (3.8%), triazophos (3.8%), acephate (2.8%), methamidophos (1.2%), isocarbophos (0.9%), dicofol (0.7%), coumaphos (0.5%), and sulfotep (0.2%). Furthermore, of the 27 detected pesticide residues, the maximum residue limits (MRLs) are priority referenced in the Chinese national standard GB/T 2763-2021^42^, followed by the Chinese regulations (MRLs of pesticides in vegetable routine monitoring in 2015)^43^, list of prohibited pesticides for legumes in China^44^, and the MRLs of pesticides set by the European Commission^45^, as shown in Table 3. Indeed, the residues for ten pesticides in 17.1% of the samples exceeded their MRLs, with difenoconazole exceeding the MRL by 380%. In addition, MRL exceedance rates were found for cypermethrin (8.5%), difenoconazole (6.4%), parathion-methyl (3.8%), chlorfenapyr (3.1%), cyfluthrin (1.4%), cyhalothrin (1.4%), pyridaben (0.9%), fenvalerate (0.3%), fenitrothion (0.2%), and fenpropathrin (0.2%). These values imply that these frequently detected pesticides were used widely and extensively in the cultivation of cowpea in the Hainan province, China. Thus, for the production and safe supply of agricultural products, the government needs to strengthen monitoring of the agricultural supply market, strictly control the sale and use of prohibited pesticides, and strengthen the training and management of sales staff in agricultural stores. It is also suggested that the rational use of these pesticides should be regulated.

**Figure 2:**
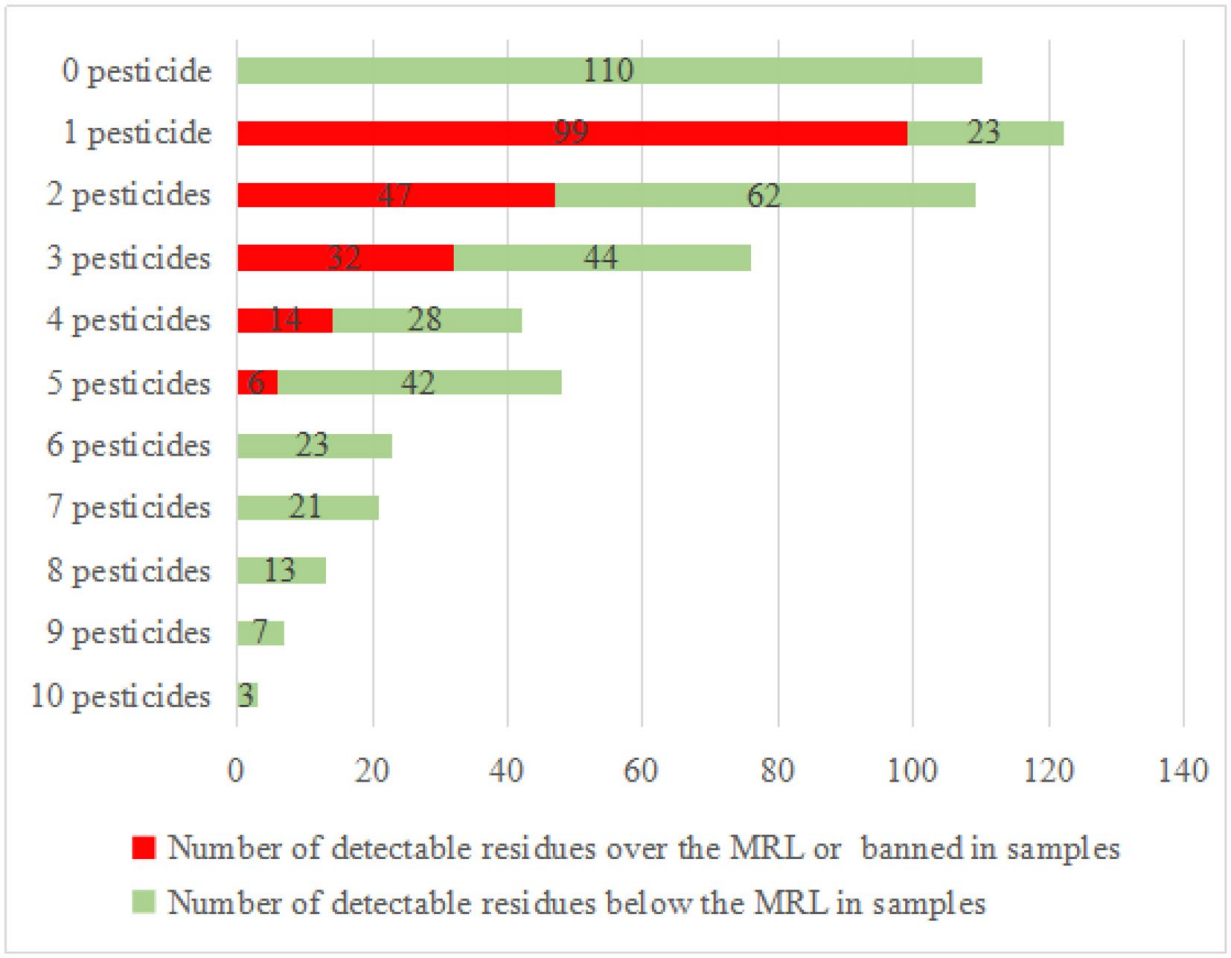
Number of detectable residues in individual cowpea samples.

As shown in Fig. 2, samples with multiple pesticide residues (two or more detected pesticide residues) accounted for 59.5% of the total number of samples, those containing one pesticide for 21.3%, and residue-free samples for 19.2%. The overall rate of samples containing multiple residues was higher than the rate of samples with no residue and a single residue, and the sample numbers decreased with the increase of pesticide residues. This finding is consistent with those from previous studies of cowpea^1,3,47^, green pepper^46^, cucumber^46^, peach^39^ and apple^47^; however, up to 10 different pesticides were detected in three samples of cowpea. Moreover, 99 of the 122 samples with one pesticide residue, 47 of the 109 samples with two pesticide residues, 32 of the 76 samples with three pesticide residues, 14 of the 42 samples with four pesticide residues, and six of the 50 samples with five pesticide residues, exceeded their MRLs (Fig. 2). This could be due to the overuse of mixture pesticides for plant protection, which can lead to major multiresidue regarding food safety^39^. Therefore, effective national food control systems, such as Good Agricultural Practices (GAP), which establish a national pesticide monitoring program that is widely accepted in most countries, are essential to protect the health and safety of domestic consumers.

### Comparison of different years

**Figure 3:**
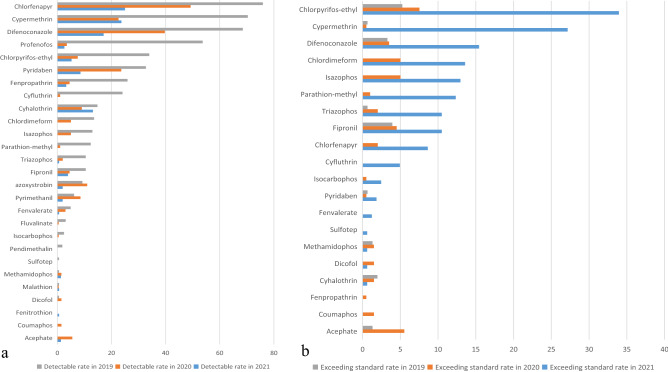
Comparison of pesticide residue detection rate and over standard rate of cowpea in different years.

A total of 574 samples of cowpea (*Vigna unguiculata* [L.] Walp) were collected, including 61 samples from 2018, 152 samples from 2019, 199 samples from 2020, and 162 samples from 2021. The samples from 2018 are relatively small and unrepresentative, and thus, they were not included in the comparison. A total of 17 pesticides were detected in 2021, while 24 pesticides were detected in 2019 and 2020 (Fig. 3a). The observed trend was that the detection rate of the same pesticide decreased each year. In addition, pesticide residues of azoxystrobin, chlorfenapyr, chlorpyrifos-ethyl, cyhalothrin, cypermethrin, difenoconazole, fenpropathrin, fenvalerate, fipronil, malathion, methamidophos, profenofos, pyridaben, pyrimethanil, and triazophos were detected from 2019 to 2021, indicating that these pesticides were all used in all the tested years. Compared to 2019, two new pesticides, acephate and coumaphos, were detected in 2020, and compared to 2020, one new pesticide, fenitrothion, was detected in 2021. However, Duan et al. reported that the most important residues of the 433 fresh cowpea samples from Hainan province in 2012 and 2013 were triazophos, carbofuran, isocarbophos, phoxim, and omethoate^3^. Our results show that the pesticide triazophos is still currently being used. This might be because in addition to the spraying of conventional pesticides, cowpea farmers may use different exploratory pesticides each year. As shown in Fig. 3b, 10 banned pesticides were detected in 2019, 11 in 2020, and five in 2021, revealing a decreasing trend in banned pesticide usage. Moreover, four pesticides (cyhalothrin, cypermethrin, difenoconazole, and pyridaben) that exceeded their MRLs were detected from 2019 to 2021, indicating that there may be excessive pesticide dosage and spraying times in cowpea cultivation.

### Comparison by region

**Figure 4:**
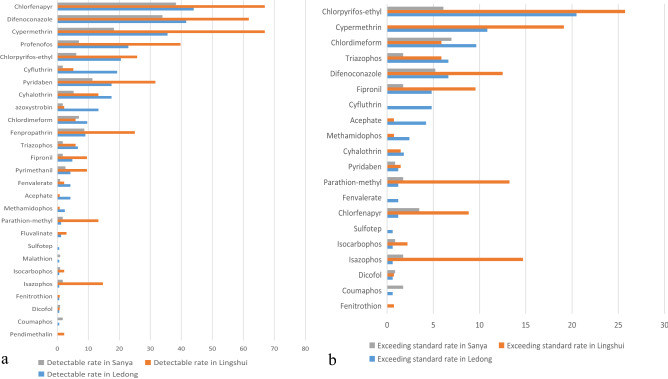
Comparison of pesticide residue detection rate and over standard rate of cowpea in different areas.

From 2018 to 2021, a total of 166 samples from Ledong, 136 samples from Lingshui, 115 samples from Sanya, 52 samples from Chengmai, 29 samples from Wanning, 40 samples from Haikou, and 36 sample from Danzhou were collected. The main winter cowpea production areas in Hainan are Ledong, Lingshui, and Sanya. Accordingly, the samples from these three areas are more relative and representative than those of the other regions, and thus, they were selected for the region comparison. A total of 26 pesticides were detected in Ledong, 24 pesticides in Lingshui, and 21 pesticides in Sanya (Fig. 4a). In addition, pesticide residues of azoxystrobin, chlordimeform, chlorfenapyr, chlorpyrifos-ethyl, cyfluthrin, cyhalothrin, cypermethrin, dicofol, difenoconazole, fenpropathrin, fenvalerate, fipronil, isazophos, isocarbophos, parathion-methyl, profenofos, pyridaben, pyrimethanil, and triazophos were all detected in Ledong, Lingshui, and Sanya, indicating that these pesticides were all used in the three main production areas. As shown in Fig. 4b, eight banned pesticides (chlordimeform, chlorpyrifos-ethyl, dicofol, fipronil, isazophos, isocarbophos, parathion-methyl, and triazophos) and three pesticides (chlorfenapyr, difenoconazole, and pyridaben) that exceeded their MRLs were detected in all three regions. This shows that some farmers use prohibited pesticides in these areas, and the sources need to be traced. Therefore, there may be excessive pesticide dosage and spraying times in cowpea agriculture in these regions.

### Dietary exposure risk assessment

Dietary exposure was used to assess the possible exposure routes and dose levels and to clarify the actual and expected exposure levels and possible harm caused to sensitive groups. The chronic hazard quotients of different populations calculated based on average pesticide residues are listed in Table 3^48^. The chronic hazard quotient of all the pesticides detected in cowpea was < 100%, indicating that the contribution of the pesticide residues in Hainan cowpea, to the risk of chronic dietary exposure, was negligible. The magnitude of the chronic hazard quotient in different groups of the same gender was consistent, and in the order (2–7-year-olds) > (8–12-year-olds) > (13–19-year-olds) ≥ (65+ -year-olds) ≥ (20–50-year-olds) ≥ (51–65-year-olds). This trend was attributed to the weight difference of the different groups and their cowpea intake. The magnitude of the chronic hazard quotient of the same population of different genders was consistent with that of female ≥ male, because of the lower weight and daily intake in females compared to those in males. The analysis revealed that dietary exposure gradually decreased with age, with children in the 2–7 age range having the highest dietary exposure. In addition, female dietary exposure was slightly higher than that of males within the same age group. A similar phenomenon has also been observed in previous studies^1,46,47^. Notably, unlike foreigners, Chinese people stir-fry vegetables before consumption, which could reduce the risk of dietary exposure^3^. Similarly, it also has been reported that blanching (5 min) followed by stir-frying (3 min) is recommended to citizens as the safest household cowpea processing method^49^. Therefore, we suggest that cowpeas should be blanched and/or stir-fried prior to consumption to reduce the risk.

## Conclusions

A QuEChERS-GC–MS/MS method for the simultaneous determination of 35 pesticides in cowpea was successfully validated. The developed method showed satisfactory recoveries and precision (70–120%, RSD < 20%) at 50, 100, and 250 μg/kg for 35 pesticides. In addition, the LOQ can meet the detection requirements of the maximum residue limits of 35 pesticides in cowpea of the European Union and other countries. A total of 574 samples of cowpea from the Hainan province of China were analyzed, and 80.8% of them tested positive for pesticides. According to the actual survey in each producing area, the possibility of the active use of restricted pesticides during production is low. However, 12 kinds of restricted pesticides were detected in the verification analysis, indicating that farmers use restricted pesticides; in this case, these sources need to be traced. Residues in 30.1% of the samples exceeded their MRLs and twelve were of banned pesticides. In addition, the forbidden pesticide triazophos was detected through the different years and regions. From the perspective of pesticide MRLS and dietary risk, the pesticide residue level of cowpea in Hainan province is not high, and the chronic dietary risk of pesticides in different genders and ages was either very low (< 3%) or within the acceptable range (< 100%). This study provides technical support for human health protection.
